# Létalité de l’insuffisance cardiaque au Centre Hospitalier Universitaire de Libreville (CHUL) et facteurs associés

**DOI:** 10.11604/pamj.2018.31.27.13259

**Published:** 2018-09-13

**Authors:** Elsa Ayo Bivigou, Mahutondji Christian Allognon, Francis Ndoume, Jean Bruno Mipinda, Emmanuel Ecke Nzengue

**Affiliations:** 1 Département de Cardiologie, Faculté de Médecine, Université des Sciences de la Santé, Libreville, Gabon; 2 Service de Cardiologie, Centre Hospitalier Universitaire de Libreville, Libreville, Gabon

**Keywords:** Insuffisance cardiaque, létalité, facteurs associés, Libreville, Heart failure, mortality, associated factors, Libreville

## Abstract

**Introduction:**

L'insuffisance cardiaque (IC) est une cause fréquente de décès en Afrique. La présente étude a pour but de déterminer le taux de létalité de l'IC et de rechercher les facteurs associés.

**Méthodes:**

Il s'agissait d'une étude rétrospective transversale réalisée dans le service de cardiologie du CHUL. Elle portait sur l'analyse de dossiers de patients hospitalisés pour IC gauche ou globale colligés de Janvier 2014 à Décembre 2016.

**Résultats:**

la létalité était de 10,3%. L'âge moyen des patients décédés (n=64) était de 57,4 ± 17 ans. Le délai moyen de prise en charge était de 15± 18 jours et la décompensation faisait suite à un écart de traitement chez 51,4% des patients décédés. L'association hypertension artérielle et diabète augmentait significativement le risque de décès (OR= 2,2 (1,2-6,6)). Les facteurs associés à la létalité étaient essentiellement: l'hypotension artérielle (OR=6,8(3,2-14,1)), l'insuffisance rénale sévère (OR=3,5(1,7-7,2)), un index cardio-thoracique supérieur à 0,7 (OR= 54,4 (15,3-193,1)), une altération sévère de la fraction d'éjection ventriculaire gauche (OR= 3,0(1,5-5,9)) et un taux élevé de NT-proBNP (OR=3,5(1,2-10,5)). La mortalité augmentait significativement avec le nombre de comorbidités. Les décès étaient dus dans 28,4% des cas à une complication extracardiaque.

**Conclusion:**

La létalité de l'IC est liée à la sévérité et à la précocité des lésions. Le retard de prise en charge et les comorbidités aggravent cette létalité. Le dépistage des facteurs de risque et l'éducation thérapeutique pourraient diminuer cette mortalité qui concerne des sujets relativement jeunes.

## Abstract (en)

**Introduction:**

Heart failure (HF) is a frequent cause of death in Africa. This study aims to determine the mortality rate of HF and to highlights its associated factors.

**Methods:**

We conducted a retrospective cross-sectional study in the Department of Cardiology at the Libreville University Hospital. It focused on the analysis of the records of patients hospitalized for left or global HF. Data were collected from January 2014 to December 2016.

**Results:**

Fatality rate accounted for 10.3%. The average age of dead patients (n=64) was 57,4 ± 17 years. Mean delay in treatment was 15± 18 days and decompensated heart failure was caused by a treatment gap in 51.4% of died patients. The association between high blood pressure and diabetes significantly increased the risk of death (OR= 2.2 (1,2-6,6)). The factors associated with mortality rate were essentially: arterial hypotension (OR=6.8 (3,2-14,1)), severe renal impairment (OR=3.5 (1,7-7,2)), cardio-thoracic index higher than 0.7 (OR= 54.4(15,3-193,1)), severe alteration in left ventricular ejection fraction (OR= 3.0(1,5-5,9)) and high NT-proBNP levels (OR=3.5(1,2-10,5)). Mortality increased significantly with the number of co-morbidities. Deaths were due to extracardiac complications in 28.4% of cases.

**Conclusion:**

Heart failure mortality is related to the severity and the precocity of the lesions. Delayed treatment and co-morbidities aggravate mortality. The identification of risk factors and a therapeutic education may reduce mortality in relatively young subjects.

## Introduction

L'insuffisance cardiaque (IC) est une maladie fréquente qui affecte 1 à 2% de la population adulte dans les pays développés et représente la troisième cause de mortalité cardiovasculaire en France [1]. Son incidence s'accroit avec le vieillissement de la population et l'avancée dans la prise en charge des pathologies prédisposant à long terme à l'IC [1]. Malgré les nombreux progrès thérapeutiques, médicamenteux et non médicamenteux, elle demeure une pathologie grave avec une mortalité à un an pouvant atteindre 40% après une hospitalisation [2]. L'Afrique subsaharienne, en pleine transition épidémiologique, n'est pas en marge. Les maladies cardio-vasculaires (MCV) y représentent la deuxième cause de mortalité [3]. L'Oganisation Mondiale de la Santé (OMS) estime qu'en 2020, la morbi-mortalité des MCV dans cette région aura doublée avec une population cible relativement jeune et un impact socioéconomique attendu [3]. Au cours de la dernière décennie, le poids important de ces affections dans les pays à revenus faibles ou intermédiaires, a conduit les différents états à mettre en place des Programmes Nationaux de Lutte Contre les Maladies Non Transmissibles. Au Gabon, le programme a été créé en 2011 et concerne essentiellement la lutte contre l'HTA et le diabète, facteurs de risque essentiels de maladies cardiovasculaires dont l'IC. Cependant, l'élaboration des stratégies de contrôle doit tenir compte des caractéristiques des populations. En effet, outre la prise en charge de ces facteurs de risque, de nombreux facteurs épidémiologiques [3], culturels [4], économiques [5] et génétiques [6] peuvent participer à l'installation et à la gravité d'une IC. Au Gabon, il existe actuellement peu de données publiées sur cette pathologie. Le Centre Hospitalier Universitaire de Libreville est l'hôpital de référence en cardiologie au niveau national. Le but de cette étude était d'y déterminer le taux de létalité liée à l'IC et de rechercher les facteurs associés au décès.

## Méthodes

Il s'est agi d'une étude rétrospective descriptive transversale à visée analytique menée au service de cardiologie du CHUL. Les données ont été colligées à partir de dossiers de patients hospitalisés pendant la période allant de Janvier 2014 à Décembre 2016. La létalité de l'IC a été déterminée à partir des dossiers de tous les patients admis pour décompensation cardiaque (DC) gauche ou globale et de ceux décédés. Le diagnostic d'IC était posé à partir des critères de la Société Européenne de Cardiologie (ESC). Pour l'analyse des facteurs associés au décès, 141 dossiers de patients sortis vivants et comportant toutes les variables analysées ont servi de groupe contrôle (patients vivants) et ont été comparés aux 64 patients décédés.

**Les variables relevées** Étaient les données sociodémographiques (âge, sexe), le délai entre le début des symptômes et la prise en charge dans le service de cardiologie, l'existence d'une cardiopathie connue ou non et le nombre d'hospitalisations antérieures pour IC. Les facteurs de risque cardiovasculaire (HTA, diabète, obésité, hypercholestérolémie, tabagisme) et les facteurs de décompensation cardiaque (surinfection broncho-pulmonaire, écart de traitement, poussée hypertensive, anémie, trouble du rythme supraventriculaire, syndrome coronarien aigu, embolie pulmonaire) ont été également répertoriés. Les cardiopathies sous-jacentes ont été classées en fonction de l'anamnèse, des données électrocardiographiques et échocardiographiques en cardiopathies hypertensive, valvulaire, ischémique, infectieuse, génétique, alcoolique, cardiomyopathie du péripartum et cardiomyopathie dilatées idiopathique.

**Les facteurs de mauvais pronostic** Connus de l'IC suivants ont été collectés: facteurs cliniques (dyspnée de stade IV de la NHYA, hypotension artérielle), facteurs biologiques (insuffisance rénale sévère, anémie sévère, hyponatrémie, dosage quantitatif de NT-proBNP), radiologiques (index cardio-thoracique), électrocardiographiques (bloc de branche gauche complet ) et échocardiographiques (altération sévère de la fraction d'éjection ventriculaire gauche, diamètre télédiastolique du ventricule gauche, hypertension pulmonaire sévère). Les pathologies associées à l'IC et les causes de décès ont été relevées. Le délai moyen de prise en charge en cardiologie a été subdivisé en 4 sous-groupes allant de 1 à 15 jours, de 15 jours à 30 jours, de 1 mois à 3 mois et de plus de trois mois. L'obésité était retenue pour un index de masse corporelle (IMC) supérieur ou égal à 30. Les troubles du rythme supraventriculaires regroupaient les fibrillations atriales, les flutters, les tachycardies jonctionnelles et atriales. Les syndromes coronariens aigus incluaient les syndromes coronariens aigus avec ou sans sus décalage du segment ST. Seules les embolies pulmonaires confirmées par angioscanner pulmonaire ont été colligées. En l'absence de coronarographie au Gabon, le diagnostic de cardiopathie ischémique était retenu en présence de facteurs de risque et d'un aspect de séquelle de nécrose à l'électrocardiogramme correspondant à une hypokinésie ou akinésie segmentaire à l'échocardiographie. La cardiopathie hypertensive était retenue en présence d'un antécédent ou d'une découverte récente d'HTA, d'une hypertrophie ventriculaire gauche électrique et d'une cardiopathie hypertrophique et/ou dilatée. L'existence d'un syndrome grippal précédant la décompensation ou d'un contexte infectieux évolutif (VIH ou autre) a fait évoquer une myocardite et la cardiopathie du péripartum concernaient les décompensations survenant à la fin de la grossesse ou dans les six mois suivant l'accouchement. En présence d'une intoxication énolique importante et en l'absence d'autre cause évidente, la cardiomyopathie alcoolique était retenue. Le seuil retenu pour l'anémie était un taux d'hémoglobine (Hb) < 10g/l. Elle était considérée comme sévère pour un taux ≤8 g/dl. Une clairance de la créatinine < 30ml/min, calculée par la formule de Cockcroft, définissait l'insuffisance rénale sévère. Un dosage quantitatif du NT-proBNP ≥10 000 pg/ml était retenu comme seuil de mauvais pronostic. Sur le plan radiologique, un index cardiothoracique (ICT) ≥7, une fraction d'éjection ventriculaire gauche (FEVG) < 30%, un diamètre télédiastolique du ventricule gauche (DTVG) > 65mm et une pression artérielle pulmonaire (PAPS) ≥60mmhg ont également été considérés facteurs de mauvais pronostic. Les causes de décès étaient classées en complications cardiaques et en complications extracardiaques.

**Les données** Ont été enregistrées et traitées à l'aide des logiciels Excel et Statview. Les variables quantitatives étaient présentées en moyenne ± écart type, les variables qualitatives en effectifs et pourcentages. En analyse bi-variée, la comparaison entre les variables qualitatives avait été effectuée à l'aide du test de Chi2. Les odds ratio (OR) ainsi que les intervalles de confiance à 95% (IC 95%) ont été calculés pour les facteurs associés à la létalité chez les insuffisants cardiaques. Une valeur de p < 0,05 a été considérée comme significative.

## Résultats

Au cours de la période d'étude, 1252 patients ont été admis en cardiologie. Parmi eux, 622 présentaient un tableau d'IC gauche ou globale, soit une prévalence hospitalière de 49,7%. Soixante-quatre patients sont décédés, ce qui représentait une létalité de l'IC de 10,3%. L'âge moyen des patients inclus était de 55,8± 17 ans avec respectivement 55,07± 18 ans et 57,4 ± 17 ans pour les patients vivants et décédés (p=0,37). Parmi les patients décédés, 28,8% d'entre eux avaient 60 ans ou moins. La prédominance masculine était nette dans les deux groupes (H/F = 1,9). Le délai moyen de prise en charge des patients était de 10,8 ± 7 jours, de 15± 18 jours pour les patients décédés et 10,76± 6 jours pour les vivants. Les sous-groupes étaient significativement différents avec respectivement pour les patients décédés et vivants 70,3% et 55% d'entre eux admis dans un délai de 1 à 15 jours suivis de 21,8% et 9,5% dans un délai de 15 jours à 30 jours (p<0,01).

**La cardiopathie** Était connue chez 46,8% des patients décédés et 25,7% des vivants (p<0,01). La décompensation cardiaque était le mode découverte de la cardiopathie dans 53,1% des cas.

**Les facteurs de risque cardio-vasculaire** Retrouvés sont représentés dans le Tableau 1. L'HTA était le facteur le plus fréquent chez les patients décédés (57,8%) et vivants (60%) (p <0,01). Le diabète, l'obésité et l'hypercholestérolémie augmentaient le risque de décès. En analyse bi variée, l'association HTA et diabète est également étroitement corrélée au risque de décès (OR=2,2[1,2-6,6], p<0,01)).

**Tableau 1:** Fréquence des facteurs de risque cardio-vasculaire dans la population d’étude

Parametres	Vivants		Décédés			
Facteurs de risque	n=141	%	n=64	%	OR IC [95%]	*p*
HTA	84	60,0	37	57,8	0,8[0,5-1,5]	0,43
Diabète	21	15,0	18	28,1	2,2[1,1-4,5]	0,03
Obésité	31	23,6	22	51,2	3,4[1,6-7,0]	<0,01
Hypercholestérolémie	2	1,4	13	25,0	23,0[5,0-86,1]	<0,01
Tabagisme	30	21,4	14	21,8	1,0[0,5-2,1]	0,91
Alcool	47	33,5	20	31,2	-	-
HTA et diabète	15	10,6	14	21,9	2,2[1,2-6,6]	0,02

**Les facteurs de décompensation cardiaque** Les plus fréquents chez les patients vivants et décédés étaient répartis respectivement comme suit dans les deux groupes: les surinfections broncho-pulmonaires (20,9% et 57,8%), les écarts de traitement (51,6% et 88,6%), l'anémie (45,7% et 28,1%) et les troubles du rythme supraventriculaires (32,7% et 22,0 %). La différence était significative entre les deux groupes pour l'anémie (p=0,01) et les surinfections broncho-pulmonaires (p<0,01). La présence d'une anémie était associée à une augmentation de la mortalité (OR=5,2 [2,1-12,2], p<0,01). La poussée hypertensive n'augmentait pas le risque de décès (OR=0,4 [0,12-0,9]). Un SCA était retrouvé chez 10,9% des patients décédés avec une mortalité de 100% dans ce groupe.

**La cardiopathie** Hypertensive était la plus rencontrée dans les 2 groupes (42,8% chez les vivants et 42,4% chez les décédés), suivie des cardiopathies valvulaires (24,3% et 20,4% respectivement), ischémiques (10,7% et 16,9% respectivement) et des cardiomyopathies dilatées idiopathiques (8,5% et 13,6% respectivement). Les autres cardiopathies étaient plus rares: myocardites (6,4% et 3,12%), alcoolique (5,0% et 0,0%) et génétique (0,0% et 1,7%). Il n'y avait pas de différence significative entre les deux groupes lorsque les prévalences de décès étaient comparées en fonction des différentes cardiopathies sous-jacentes (p=0,27).

**les facteurs de mauvais pronostic** Recensés figurent dans le Tableau 2. Sur le plan clinique, la dyspnée de stade IV et l'hypotension artérielle étaient plus fréquentes chez les patients décédés (p <0,01). La mortalité augmentait en présence de ces deux facteurs (p<0,01). Sur le plan biologique, le taux moyen de NT-proBNP chez les patients inclus était de 7176 ± 7121 pg/ml, de 9509pg± 8798 pg/ml chez les patients décédés et de 6251 pg/ml± 6184 pg/ml chez les vivants. Le taux moyen d'hémoglobine était de 10,7 ± 2g/dl, 9,9 ± 2,4g/dl chez les sujets décédés et 11,1 ± 1,7 g/dl chez les sujets vivants. Une insuffisance rénale sévère était retrouvée chez 56,4% des patients décédés et 43,6% chez sujets vivants. Une anémie sévère, une hyponatrémie et une altération sévère de la fonction rénale étaient les facteurs biologiques corrélés au risque de décès (p<0,01) (Tableau 2).

**Tableau 2:** Facteurs pronostiques cliniques et paracliniques associés au risque de décès

Paramètres	Vivants		Décédés			
	n=141	%	n=64	%	OR [IC95%]	p
**Facteurs cliniques**						
Dyspnée de stade IV	24	17,1	40	62,5	8,1[4,1-5,7]	<0,01
Hypotension artérielle	15	10,8	29	45,3	6,8[3,2-14,1]	<0,01
**Facteurs biologiques**						
Hb ≤8g/dl	9	6,7	16	21,2	5,1[2,1-12,4]	<0,01
NTproBNP ≥ 10 000 ng/ml	9	15,5	9	39,1	3,5[1,2-10,5]	0,03
Clairance créatinine <30ml/min	17	12,8	22	34,8	3,5[1,7-7,2]	<0,01
Hyponatrémie	25	22,3	25	51,0	3,6[1,8-7,4]	<0,01
**Facteurs radiologiques**						
ICT > 0,7	3	2,3	30	56,6%	54,4[15,3-193,1]	<0,01
Hypertension artérielle pulmonaire sévère	13	9,9	9	21,4	2,4[1,0-6,1]	0,06
Altération sévère FEVG	46	34,2	28	60,8	3,0[1,5-5,9]	<0,01
Diamètre télédiastolique du VG ≥65mm	41	30,8	15	35,7	1,3[0,6-2,9]	0,06
**Facteurs électrocardiographiques**						
BBG complet	14	11,0	9	15,0	1,4[0,6-3,5]	0,30
TVNS	1	0,73	0	0,00	1,7[0,9-3,4]	0,14
BBG complet et FEVG<30%	6	4,9	6	15,6	3,6[1,1-11,3]	0,03

**Sur le plan radiologique**, L'ICT moyen était de 0,71 ± 0,1 chez les décédés et 0,61± 0,1 chez les vivants. Un index > 0,7 augmentait le risque de décès (p<0,01). En échocardiographie-doppler, la FEVG moyenne était de 29%±13 et de 36,2 % ± 15 chez les décédés et les vivants respectivement. Une altération sévère de la FEVG était retrouvée chez 60,9% des patients décédés et 34,3% des vivants. Le DTVG moyen était de 62,5±8,7 mm chez les décédés et de 59,18±9,6 mm chez les vivants. Le risque de décès n'était pas associé à ce diamètre (Tableau 2). Une PAPS > 65 mmHg était retrouvée chez 21, 4,% des patients décédés et 9,9% des patients vivants. La présence d'une hypertension artérielle pulmonaire sévère augmentait le risque de décès (p<0,01) (Tableau 2).

**A l'électrocardiogramme,** Un bloc de branche gauche (BBG) complet était décrit chez 15% des patients décédés et 10,3% des vivants. Le BBG complet isolé n'était pas lié aux décès (p =0,31). L'association altération sévère de la FEVG et BBG complet augmentait le risque de létalité (OR =3,6[1,1-11,3])

**Les pathologies associées** à L’IC sont présentées dans le Tableau 3. Chez les patients diabétiques, le diabète déséquilibré était plus fréquent chez les patients décédés comparativement aux vivants (p<0,01). Cette comorbidité augmentait le risque de décès (OR=16 [3,0-64,8], p<0,01)). Parmi les patients décédés, 51,5% d’entre eux avaient une comorbidité, 12,5% avaient deux comorbidités et ces fréquences étaient respectivement de 21% et 1,4 chez les vivants (p<0,01). Les autres comorbidités sont décrites dans le Tableau 3. Le risque de décès augmentait proportionnellement au nombre de comorbidités avec un OR de 3.7 [3,9-16,2] en cas d’une seule comorbidité et un OR de 20,4 [4,1-102]) en cas d’au moins deux comorbidités. Le décès était lié à une complication extracardiaque dans 26,5% des cas dont 6 (9,3%) complications hémorragiques graves, 3 (4,6%) accident vasculaire cérébraux et 3 (4,6%) sepsis.

**Tableau 3:** Fréquence des comorbidités associées à l’IC en fonction de l’évolution

Paramètres	Vivants		Décédés		
Comorbidités	n=141	%	n=64	%	*p*
Diabète déséquilibré	7	33,3	16	88,8	<0,01
Insuffisance rénale terminale dialysée	0	0,00	4	6,2	<0,01
Antécédents d’AVC	8	5,7	6	9,3	0,33
Pathologie hépatique évoluée	0	0,0	5	7,8	<0,01
VIH	5	3,6	1	1,6	<0,01
Bacillose	1	0,7	2	3,1	<0,01
Néoplasie	0	0,0	3	4,7	<0,01

## Discussion

L'étude THESUS-HF, regroupant neuf pays d'Afrique subsaharienne, a permis d'avoir des données sur l'ampleur de l'IC dans cette région du globe et l'impact socio-économique défavorable attendu [7]. Au Gabon, l'épidémiologie de l'IC est mal connue. Cette étude est la première à rapporter la prévalence et le taux de létalité de l'IC en milieu hospitalier gabonais. Elle a été réalisée au Centre Hospitalier Universitaire de Libreville, hôpital de référence en Cardiologie au niveau national. Cette structure publique accueille les patients de la capitale Libreville ainsi que ceux référés des hôpitaux et dispensaires des différentes provinces. Les résultats apportent donc des données préliminaires importantes.

La prévalence hospitalière de l'IC était de 49,7%. Le taux de létalité était de 10,3%. Ce taux varie dans les différentes séries hospitalières africaines avec 11,86% à Lomé [8], 9,03% à Yaoundé [9] et 10,1% à Kano au Nigéria [10]. Il reste néanmoins supérieur à ceux des pays à revenus plus élevés tels que le Maroc (6,1%) [11] et la France (7,5%) [1]. De nombreux facteurs, parmi lesquels le retard de prise en charge, pourraient expliquer cette surmortalité de l'IC dans nos régions. En effet, le délai moyen prise en charge des patients décédés allait au-delà de 15jours. Ce retard, également souligné dans d'autres études [12], est parfois lié aux contraintes financières limitant l'accès aux soins dans nos régions. Le coût de l'hospitalisation, souvent à la charge du patient, a été estimé à 6,4 fois le SMIG en 2012 au TOGO [5]. L'âge moyen des patients décédés était de 57,4 ans tel qu'au Togo [12] et en Tanzanie (62 ans) [13]. Ce qui contraste avec les pays développés dont la France où, selon les registres nationaux, l'âge moyen de décès est de 86,4 ans. Les décès dits prématurés en France (survenant chez les moins de 65ans) ne représentent que 3,5% des patients [1] alors que près d'un tiers des décès dans la présente étude surviennent avant 60 ans. L'enjeu socio-économique est donc différent entre les pays développés et l'Afrique subsaharienne où les décès par IC concernent une population jeune et active.

L'HTA est le facteur de risque cardiovasculaire le plus fréquent dans la plupart des populations noires africaines [7-10, 13-15] mais aussi Afro-américaines (37% de prévalence) [16]. Cela constitue un véritable problème de santé publique vue les particularités de l'HTA sur ce terrain. En effet, le sujet noir présente des formes sévères, souvent résistantes et sources de complications cardiaques précoces [6]. Toutefois, il n'y avait pas de corrélation entre les antécédents d'HTA et le risque de décès au CHUL (p=0,77). Ce résultat pourrait s'expliquer par le fait que l'HTA aurait un effet protecteur sur la mortalité par IC à 30 jours avec un impact qui deviendrait délétère à plus long terme [16]. L'association HTA et diabète augmentait le risque de décès. La prévalence hospitalière du diabète à Libreville était estimée à 2% en 2015 [17] et une HTA était associée chez 40,7% d'entre eux [18]. Le dépistage précoce par des campagnes de sensibilisation et le contrôle optimal de ces deux facteurs de risque cardio-vasculaire majeurs permettrait de diminuer la mortalité liée à l'IC au Gabon.

Un écart de traitement était rapporté chez 51,6% des patients décédés. La non compliance au traitement chez le sujet noir africain a été étudiée en Côte d'ivoire [4] et en Tanzanie [13]. Il apparait qu'elle n'est pas toujours liée au faible niveau social, au coût ou à l'existence d'une couverture sociale. En effet, le Gabon dispose actuellement d'une couverture médicale nationale qui a permis de diminuer les coûts de traitement mais la compliance reste mauvaise. Outre le recours à la médecine traditionnelle [4], le niveau d'éducation thérapeutique, jugé bas dans d'autre pays d'Afrique noire [4, 13], explique ces écarts de traitement. Ce constat souligne la nécessite de mettre en place, au sein des hôpitaux des programmes d'éducation thérapeutique qui pourraient augmenter l'adhésion au traitement (niveau de grade I dans les Recommandations Européennes de prise en charge de l'IC [19, 20]). Une prise en charge multidisciplinaire incluant des diététiciens serait bénéfique pour l'amélioration de l'observance du régime hyposodé. En effet, la surconsommation de sel est décrite chez le sujet noir [6, 21]. Les surinfections broncho-pulmonaires, fréquentes dans d'autres études [14], étaient associées au risque de décès. La vaccination antigrippale et antipneumococcique des insuffisants cardiaques, recommandée par la Société Européenne de Cardiologie [19, 20], semble difficilement applicable dans nos régions du fait du coût et de l'épidémiologie particulière. La mortalité liée aux SCA, bien que faible, souligne l'importance de développer les techniques de revascularisation actuellement indisponibles au Gabon.

L'anémie, fréquente et multifactorielle dans l'IC [14, 22, 23] est un facteur péjoratif à tous les stades de l'affection [3]. Elle entraine une diminution de la capacité fonctionnelle d'effort et est souvent rapportée comme facteur indépendant de mortalité [13].Toutefois, sa prévalence dans l'IC varie d'une série à l'autre [10, 13, 14, 23] en fonction de la définition retenue. En effet, l'Organisation Mondiale de la Santé définit l'anémie comme un taux d'Hb< 13g/dl chez l'homme et 12g/dl chez la femme. Cette définition est difficilement applicable dans l'IC du fait de l'hémodilution entrainant une diminution de l'hémoglobine. L'usage de l'hématocrite est de ce fait associé à la définition de l'anémie dans l'IC. Par ailleurs, le sujet noir présente des particularités. En se basant sur la définition de l'OMS, Gretchen [24] rapporte un taux moyen d'hémoglobine de 11,9g/l dans la population adulte d'Afrique Centrale avec près de 2% des sujets qui présentent une anémie sévère inférieure à 7g/dl. Cette anémie est d'origine multifactorielle (carence martiale et vitaminique, parasitaire, hémoglobinopathies). Avec un taux d'hémoglobine défini à moins de 10g/dl , elle multiplie par 5 le risque de décès dans cette série. Les Recommandations Européennes [19] préconisent la recherche systématique d'une carence martiale associée à l'IC, ce qui n'est pas toujours réalisé en contexte africain. Sa correction par l'administration orale ou injectable de sulfate ferreux est devenue une des cibles du traitement de l'IC pour améliorer la capacité fonctionnelle d'effort. L'effet sur la mortalité n'est cependant pas toujours démontré.

La cardiopathie hypertensive prédomine ailleurs en Afrique subsaharienne. Un dépistage précoce de l'HTA et une prise en charge adaptée pourraient éviter l'évolution insidieuse vers l'IC qui est souvent le mode de découverte de cette pathologie [10, 12, 13]. Contrairement au Maghreb [11] et en Europe [1], la cardiopathie ischémique est moins fréquente. Elle est probablement sous-estimée dans nos régions du fait de la faiblesse du plateau technique (absence de salle de coronarographie) et le nombre important de cardiomyopathies dites indéterminées (12,5% au CHUL et 5,9% à Lomé [12]). Seuls 3,12% des patients décédés avaient un diagnostic de myocardite à VIH. Ce qui confirme la transition épidémiologique dans nos régions avec le recul des pathologies infectieuses et l'essor des affections liées à l'athérosclérose. L'évaluation de la sévérité et du pronostic fait partie du bilan initial de toute IC selon les recommandations de la HAS en France [25]. Quelques études africaines se sont également intéressées à la recherche de ces facteurs pronostics. L'hypotension artérielle augmente de huit fois le risque de décès dans cette série et quatre fois en Ouganda [14]. Elle témoigne du bas débit cardiaque et peut empêcher l'utilisation de doses optimales des traitements usuels de l'IC (inhibiteurs de l'enzyme de conversion, béta bloquants, dérivés nitrés). La dyspnée de stade IV était également associée au risque de décès (OR 8,1). Ce mode de présentation est souvent rapporté par d'autres auteurs [11, 12, 15] en Afrique sub-saharienne et rend compte du retard de prise en charge des patients.

Sur le plan biologique, le BNP ET NT-proBNP sont les biomarqueurs pronostiques les plus puissants et les plus utilisés dans l'IC. Ils témoignent de l'élévation des pressions de remplissage et du degré de dilatation ventriculaire. Un taux de NTproBNP supérieur à 10 000pg/ml dans cette série multipliait par 3,5 le risque de décès. Le taux moyen de NT-proBNP des patients décédés était de 9509pg/ml. Il est près de deux fois supérieur au seuil de mauvais pronostic rapporté dans une étude américaine qui était de 5180 pg/ml [26]. Peu de séries en Afrique permettre de comparer nos résultats à une population de race noire décédée d'IC. Par contre, une étude américaine avait mis en évidence des taux de NT-proBNP plus élevés chez les Afro-américains comparés aux sujets de race blanche [27]. Ce qui peut expliquer nos résultats. Aussi, les taux sanguins de ce marqueur sont augmentés en présence d'une insuffisance rénale, qui concernait 56,4% des patients décédés et 43,9% des vivants. Par ailleurs, ces taux reflètent la sévérité des lésions échocardiographiques observées. L'insuffisance rénale, facteur indépendant de mortalité dans l'IC [22], est multifactorielle. Elle peut être une complication précoce de l'HTA chez le sujet noir [6] et est alors associée à l'IC. Elle peut également compliquer une IC et témoigner du bas débit rénal. La FEVG moyenne des patients décédés (34%) peut ainsi expliquer la proportion d'insuffisance rénale sévère dans cette série. L'ICT moyen retrouvé est proche de celui d'autres auteurs [9, 23]. Supérieur à 0,7, il augmentait significativement le risque de décès. Dans l'étude de Kearney [28], il était associé au risque de mort subite. Ce paramètre simple, obtenu par la radiographie thoracique de face, peut être facilement utilisable dans notre contexte socio-économique pour évaluer le pronostic d'une IC.

La décompensation cardiaque était le mode de révélation de la cardiopathie chez 53,1% des patients décédés. Les lésions échocardiographiques évoluées témoignent du retard diagnostique, tel que décrit à Lomé [12], mais aussi de la sévérité des lésions chez le sujet noir [14]. La dilatation ventriculaire gauche moyenne est plus importante (62 mm dans cette étude, 61 mm à Lomé [12]) comparée aux populations caucasiennes (56mm) [29]. La dysfonction ventriculaires gauche sévère est fréquemment rapportée dans les séries africaines [10-14]. Elle multipliait par trois le risque de décès au CHUL et par sept en Ouganda [14]. L'augmentation de la mortalité dans le groupe associant altération sévère de la FEVG et la présence d'un BBG complet souligne l'intérêt de la mise en route de programmes de resynchronisation au Gabon. Bien que l'effet sur la mortalité ne soit pas démontré, il permet une amélioration de la capacité fonctionnelle des patients insuffisants cardiaques avec un QRS large.

L'IC est une maladie chronique grave souvent associée à des comorbidités. Le nombre de comorbidités est significativement corrélé à la mortalité dans cette étude. En effet, la présence de ces comorbidités complique la prise en charge et aggrave le pronostic de l'IC [22]. Le traitement optimal est parfois limité en présence d'une atteinte rénale ou hépatique, nécessitant une adaptation des posologies et exposant aux risques de surdosage. Le traitement des pathologies associées augmente également le risque d'interactions médicamenteuses. Le décès de l'insuffisant cardiaque, surtout en présence de comorbidités, survient parfois des suites d'une complication extracardiaque telle que chez 26,5% des patients inclus. La complexité de la prise en charge de l'IC nécessite parfois une approche pluridisciplinaire. Cette étude présente des limites notamment le caractère rétrospectif qui n'a pas permis l'analyse d'autres facteurs pronostiques de l'IC (fréquence cardiaque, dysfonction VD, CRP, acide urique, taux de triglycérides et de cholestérol etc…) et le fait de n'avoir pu analyser que 141 dossiers de patients vivants. Toutefois, le calcul de la taille de l'échantillon, basée sur la prévalence des décès dus à l'IC (estimée à 0,10%) a estimé ce dernier à 139. Dans la présente étude, 206 patients ont été inclus. Une étude étendue à l'ensemble des structures sanitaires publiques pourrait permettre d'obtenir des données nationales.

## Conclusion

La létalité de l'IC concerne des patients relativement jeunes. L'ensemble des facteurs de mauvais pronostique cliniques et paracliniques témoignent du retard de prise en charge des patients. Les atteintes cardiaques sont souvent d'emblée sévères et associées à de nombreuses comorbidités. Le dépistage et la prise en charge précoce des facteurs de risque, notamment l'HTA et le diabète, par des programmes nationaux étendus à l'ensemble du territoire pourrait diminuer cette létalité liée à l'IC. Un accent particulier devra être mis sur l'éducation thérapeutique pour améliorer l'adhésion des patients au traitement.

### Etat des connaissances actuelles sur le sujet

- L'insuffisance cardiaque est une cause fréquente de morbidité et de mortalité en Afrique subsaharienne. Elle affecte principalement les jeunes. La prise en charge des patients est souvent retardée.

### Contribution de notre étude à la connaissance

- Cette étude fournit les premières données sur la mortalité par insuffisance cardiaque au Gabon;
- La présence de comorbidités augmente considérablement le risque de décès;
- La forte association entre les modifications de traitement et la décompensation cardiaque souligne la nécessité de mettre en œuvre des programmes d'éducation thérapeutique.

## Conflits d’intérêts

Les auteurs ne déclarent aucun conflit d'intérêts.
